# The collection of speech data for the assessment of cognition remotely: Balancing ethical and practical challenges

**DOI:** 10.1002/dad2.70341

**Published:** 2026-06-08

**Authors:** Stina Saunders, Fasih Haider, Alyssa M. Lanzi, Brian MacWhinney, Davida Fromm, Chia‐Ju Chou, Yi‐Chien Liu, Ya‐Ning Chang, Sarah Gregory, Craig Ritchie, Ioulietta Lazarou, Spiros Nikolopoulos, Ioannis Kompatsiaris, Samuel O. Danso, Graciela Muniz‐Terrera, Juan Rafael Orozco‐Arroyave, Paula Andrea Perez‐Toro, Raquel Rangel Cesario, Manuel Cesario, Saturnino Luz

**Affiliations:** ^1^ Usher Institute, School of Population Health Sciences The University of Edinburgh Edinburgh UK; ^2^ Department of Communication Sciences and Disorders University of Delaware Newark Delaware USA; ^3^ Department of Psychology Carnegie Mellon University Pittsburgh Pennsylvania USA; ^4^ Department of Neurology Cardinal Tien Hospital New Taipei City Taiwan; ^5^ Miin Wu School of Computing National Cheng Kung University Tainan City Taiwan; ^6^ Centre for Clinical Brain Sciences University of Edinburgh, Edinburgh BioQuarter Edinburgh UK; ^7^ School of Medicine University of St. Andrews St. Andrews UK; ^8^ Centre for Research and Technology Hellas Information Technologies Institute, Thermi Thessaloniki Greece; ^9^ School of Computer Science and Engineering University of Sunderland Sunderland UK; ^10^ School of Allied Health Sciences University of Cape Coast Cape Coast Ghana; ^11^ Heritage College of Osteopathic Medicine Ohio University Athens Ohio USA; ^12^ GITA Lab Universidad de Antioquia Antioquia Colombia; ^13^ Pattern Recognition Lab Friedrich‐Alexander‐Universität Erlangen‐Nürnberg Erlangen Germany; ^14^ Magdala Academy São Paulo Brazil; ^15^ Medicina Uni‐FACEF São Paulo Brazil

**Keywords:** Alzheimer's disease, brain health, detection of cognitive decline, outcome measures, speech biomarkers

## Abstract

Speech biomarkers could form a critical step in improving the accessibility, scalability, and early detection of Alzheimer's disease and related dementias. However, ethical and practical challenges remain across regulatory and cultural contexts. In this paper, we briefly review the challenges in adopting speech biomarkers, relate our experiences globally to recent advances in the neurodegenerative field, and consider how speech assessments could be integrated into clinical care. Insights from high‐ and low‐ and middle‐income countries (Taiwan, Ghana, Colombia, Brazil, Greece, UK, and US) demonstrate the potential impact of speech technology. While there are common benefits, risks, incentives, and hurdles, many aspects are specific to country or region. There is a need for more speech data sets (particularly in languages other than English), standardization in data collection and analysis, stronger collaboration between machine learning and neurodegenerative disease experts, unified privacy regulation, and finally, a consensus on the clinical interpretation of speech biomarker data.

## Highlights

- Remote speech‐based biomarkers have potential as scalable tools for early neurodegenerative disease detection across varied health‐care systems globally.
- We present insights from seven countries highlighting both shared benefits and region‐specific hurdles to implementation and equity of speech‐based biomarkers.
- Clinical adoption depends on validation of norms, clinician training, and culturally adapted tools.
- Global disparities in available large language models and privacy regulation hinder development and deployment of speech biomarkers.
- Remote assessments can increase clinical reach in rural populations, supporting more inclusive brain health care worldwide.

## 1 BACKGROUND

Neurodegenerative brain diseases impact areas of speech long before overt clinical symptoms of Alzheimer's disease (AD) and related dementias (ADRD) appear, influencing aspects such as richness of vocabulary, fluency, and acoustic features of speech.^1^ Consequently, speech‐based biomarkers have been proposed as a cost‐effective method for the early identification, prognosis, and management of ADRD.^2^ The primary appeal of speech biomarkers lies in their ease of use for seldom heard populations and longitudinal monitoring across diverse cohorts and diseases.^3^, ^4^

Recent research has accelerated speech‐based biomarkers as indicators of preclinical, prodromal, and symptomatic neurodegenerative disease. Research in this area is increasingly shifting from single‐feature acoustic analyses toward integrative models incorporating both linguistic and paralinguistic features, reflecting the fact that neurodegeneration alters multiple layers of speech production.^5^ While cognitive tests administered in clinical environments have included speech tasks such as category naming, text reading, and picture description, speech data collection has become more feasible in naturalistic settings. In one recent multi‐day remote tablet‐based study of cognitively unimpaired individuals, for instance, > 90% of participants adhered to the study protocol, and averaged acoustic features showed good test–retest reliability and high usability scores.^6^ The same study observed differences in specific acoustic markers (e.g., pause‐to‐word ratio) between amyloid beta (Aβ)‐positive and ‐negative participants, although the higher pause‐to‐word ratio in Aβ‐positive participants did not remain significant after correction for multiple comparisons.

Similarly, Cho et al.^7^ developed an automated language processing framework that distinguished mild cognitive impairment (MCI) from no impairment with high accuracy using naturalistic narrative speech. Their approach uses transformer‐based language models trained on age‐matched data, to detect subtle lexical–semantic shifts indicative of early cognitive decline (Supporting Information).

This growing body of evidence supports the potential clinical relevance of speech biomarkers for early‐stage AD, though many key aspects of their reliable use remain challenging, including determining the etiology of decline. This paper discusses the utility of speech biomarkers in the detection, monitoring, and management of clinical syndromes associated with ADRD in a global context, and explores the value, risks, and future applications of using speech biomarkers to remotely monitor cognition and brain health.

## 2 THE MAIN ADVANTAGES AND DISADVANTAGES OF SPEECH BIOMARKERS

Assessment of neurodegenerative disease typically requires testing in person, conducted by trained and qualified team members. In contrast, remote assessments offer a valuable alternative for both assessment and ongoing care in ADRD,^8^ reducing travel burden for the person and care partner and the need for specialist staff time. Additionally, speech biomarkers and telemedicine widen accessibility to seldom heard groups and increase scalability of both early detection and monitoring of cognitive function. For instance, the DementiaBank protocol in the United States was specifically designed to be delivered via telehealth and enables reaching participants in remote rural locations as well as population groups who are typically underrepresented in research,^9^ although noise‐reduction and compression algorithms associated with different platforms may impact the types of speech acoustics analyses that can be validly and reliably extracted. Moreover, the use of everyday digital devices in remote monitoring enables frequent, ecologically valid data collection.

The argument in favor of speech‐based biomarkers in AD lies in their sensitivity to early cognitive changes as well as the possibility of detecting and monitoring wider well‐being, such as mood disorders^10^ and apathy,^11^ which often overlap with AD. Speech biomarkers are an area with rapidly growing evidence around their utility in both research and clinical contexts,^12^ demonstrating a demand for remote, scalable assessment methods in neurodegenerative disease research and clinical practice. The remote capability is especially valuable in countries where there are difficult‐to‐access regions, but also ongoing internal conflict where access is restricted by guerrilla activity. Additionally, in countries like Ghana, where there are strong cultural beliefs against blood‐based approaches to screening,^13^ non‐invasive speech‐based biomarkers present a possibly more appropriate and acceptable alternative for early detection and monitoring of cognitive decline. Further, in Ghana, Colombia, Brazil, and many other low‐ and middle‐income countries (LMICs), remote data collection and telehealth are becoming increasingly feasible and commonly used.^14^ This potential for broadening access aligns with the targets of the World Health Organization (WHO)’s global action plan for public health responses to dementia, particularly in regard to risk reduction and diagnosis, treatment, and care, including the target that at least 50% of countries achieve a diagnosis rate of at least 50%, which remains far from being met.^15^

Finally, speech‐based assessments that predict amyloβ status^6^, ^16^, ^17^ would allow non‐invasive screening of AD pathology and eligibility assessment for anti‐amyloid antibody treatments.^18^ On these grounds, speech biomarkers appear to be a critical component in addressing the multifaceted challenges in managing ADRD.

Despite these advantages, there are several concerns associated with speech biomarkers, including cultural insensitivity,^19^ demographic biases,^20^ privacy concerns,^21^ and the lack of interdisciplinary collaboration between experts in statistical methods, machine learning/artificial intelligence (AI), and clinical neuroscience. Furthermore, there is no consensus around how to standardize data acquisition protocols, such as the volume and variety of speech data needed to ensure the accurate tracking of pathological changes.^12^

With more than half of the world's population speaking more than one language,^22^ it is critical to derive speech norms for non‐European languages^23^ as well as adapt and update language technology components and models that apply in bilingual and multilingual settings, including changes in language over time. While new technologies have enabled data collection on relatively large scales, there are still several technological challenges, such as accounting for bilingualism, in which frequent language switching in multilingual households complicates data collection and modeling. While some of these challenges could be tackled, in principle, by incorporating such covariates into predictive models, doing so requires careful design.

The main limitation in studies analyzing languages other than English is that most language models, notably large language models (LLMs), have been trained mostly on English speech data, and very few LLMs trained primarily in other languages exist.^24^ Other kinds of machine learning and signal processing models used in typical speech processing systems, such as acoustic feature extractors, automatic speech recognition, parsers, and so forth, also struggle to cope with low‐resource languages. This is also true of foundation models that target acoustic data,^25^ for which specific sounds of certain languages are often not adequately modeled due to data scarcity. For instance, our experience in Taiwan illustrates this issue: we have observed that machine learning models trained on limited datasets in Mandarin Chinese language may yield a high rate of false positive cases when applied to broader populations, risking clinical mismanagement of these false positive cases. Furthermore, for languages such as Taiwanese or Twi (spoken in Ghana) computational methods for automatic speech recognition and language analysis are incompletely developed. Consequently, specific lexical units and morphosyntax phenomena in these linguistic contexts are not appropriately understood in the context of ADRD.

## 3 PROTECTING PERSONALLY IDENTIFIABLE INFORMATION AND PRIVACY

It is recognized that an audio recording may carry personal information that the speaker may not intend or expect to disclose.^26^ In the context of ADRD, speech data may incorporate acoustic speech features like pitch (how high or low a speaker's voice sounds), jitter (inconsistencies or wobbles in the voice), articulation rate (speed of speaking), phonemic identifiability (the probability of a certain phoneme being correctly pronounced), and semantic coherence (words making sense),^12^, ^27^ making speech data personally identifiable information, with concerns around data storage, sharing, and the risk of re identification.^3^

Unlike structured numeric data, making speech data anonymous can be complex and resource‐intensive, often requiring machine learning expertise, staff time, and appropriate programs. Some techniques used to protect the privacy of speech data include: (1) encrypting audio files for storage and transfer; (2) splitting data into random components, each processed independently to reduce the risk of privacy leakage; (3) learning data representations, such as through auto‐encoder technologies that remove identifiable information;^28^, ^29^ (4) transfer learning, which consists of creating models that incorporate information from different locations where data collection was performed, that is, only transferring locally generated parameters without sharing any recording among different hospitals or research centers;^30^ (5) voice transformation to alter the person's acoustic features while preserving clinically relevant speech characteristics; and (6) differential privacy, which introduces statistical noise to the dataset.^31^

Although privacy concerns can be addressed to a certain extent with the use of privacy‐preserving machine learning techniques, the broader challenge lies in the limited regulation governing large‐scale data acquisition. For instance, current privacy legislation, which is limited to more traditional data protection principles such as minimizing the amount of data collected and informed consent, based on the global Fair Information Practices (FIPs), may not sufficiently consider the depth of information that can be derived from speech data.^32^

There is no global standard for speech data governance, and country‐ and region‐specific regulations vary considerably. In Latin American countries, while privacy regulations exist,^33^ they are generally more lenient than those in the EU. Furthermore, the application of regulations can vary significantly across different nations and specific contexts, particularly when dealing with sensitive information such as genetic data. In Brazil, the data protection legislation (“Lei geral de proteção de dados” [LGPD]) was introduced in 2018 and regulates rights and access to sensitive information. There has been vigorous debate since then on the implications of the LGPD to AI research, particularly in health technology.^34^ In Colombia, which hosts one of the largest populations with genetic early onset AD,^35^ privacy concerns are particularly acute. Although initiatives like RedLat^36^ and BrainLat^37^ have advanced collaborative dementia research across Latin America, data protection remains a pressing concern in cross‐border collaborations due to the need to navigate individual countries’ privacy regulations.

A proposed solution is the increasing use of trusted research environments (TRE), that is, platforms that both store and enable analysis of research data, negating the need for data to be transferred out of the secure platform and allowing access to users within the same platform.^38^ Examples include the Alzheimer's Disease Data Initiative's (ADDI) online platform (https://www.alzheimersdata.org/), the AD Workbench (https://www.alzheimersdata.org/ad‐workbench) and DATAMIND (https://datamind.org.uk).

## 4 LEGAL, INSURANCE, AND CAPACITY CONCERNS IN LONGITUDINAL MONITORING

The collection and longitudinal use of speech data in neurodegenerative disease present complex issues around data privacy and anonymity, consent in how speech data are used, and who the data are shared with. In the EU and UK, frameworks such as the General Data Protection Regulation (GDPR) necessitate strict regulatory compliance, including clear consent for speech data collection. In Brazil, the LGPD defines a national framework similar to the GDPR. While there is no federal law like the GDPR in the United States, personal information handled in connection with speech and language services conducted in health‐care settings is covered by the Health Insurance Portability and Accountability Act (HIPAA) as well as state laws around data protection. This situation is similar in countries like Colombia, where the regulation is not clear and most responsibility for patients’ privacy and data rests on the Research Ethics Committees’ criteria.

While there is no current precedent for insurance companies to access speech data, evidence around individuals’ preferences in sharing other types of AD biomarker results suggests there are concerns around stigma and discrimination with regard to sharing AD assessment results.^39^ It remains a gray area how much individuals would be expected or legally obliged to disclose to insurance companies regarding speech‐based findings on health status, and how, if at all, insurance companies would be able to interpret results from novel AD biomarker assessments.

There is no consensus in the literature on how to involve participants in longitudinal speech assessments when cognitive decline may impact capacity over time, raising ethical questions about consent and ongoing participation in the AD population. In systems such as those in Scotland, ascertaining and recording ongoing assent at each study visit aligns with both the Adults with Incapacity (Scotland) Act, 2000^40^ and Good Clinical Practice guidelines^41^ even after capacity diminishes. However, enrolling individuals in research when there is diminished capacity to consent even at study entry can pose challenges, particularly when communicating about complex data protection issues that should be considered prior to study entry. Designing clear communication aids around what speech data will be collected, for what purpose, and what safeguards are in place, to support participant and care partner decision making are key. Furthermore, partnering with people with a lived experience to design the study and participant‐facing materials can be hugely beneficial when dealing with digital technologies.^42^

We argue that the decision to enroll someone who may lack capacity to consent should prioritize the individual's past wishes, personality, and identity—even if those wishes may not align with what others perceive as “best interest” for them in a generic sense.^43^ This approach aligns with the underpinning system of organ donation, in which the person makes their personal wishes known before any need for such health‐related decisions arise. Indeed, while not yet in common use globally, the organ donor type approach could be operationalized through advance research directives through which the person in early stages of ADRD gives explicit directives regarding future research participation when they still have capacity.^44^ Efforts in this area should ensure directives are as fully informed and as inclusive as possible for potential future uses, accounting for emerging biomarkers, such as speech, alongside fast‐moving technological leaps in digital and remote data collection.

## 5 BARRIERS TO INCLUSION OF DIVERSE POPULATIONS

While remote assessment methods have the potential to offer more equitable health care across diverse populations, there are significant challenges in bringing these scientific advances to global communities. Cultural sensitivity is particularly crucial when engaging senior populations with varying levels of health literacy, who may hold differing perceptions of medical technology, shaped sometimes by cultural beliefs, which impact participation in clinical studies. For instance, in Taiwan, a rise in online scams and cybersecurity concerns has led to skepticism toward participating in trials and the use of new technologies. Additionally, socioeconomic disparities pose a significant obstacle, with lower income groups being less likely to engage with advanced technologies. Our experience with DementiaBank in the United States suggests that online platforms, such as Zoom, and community outreach may yield more trusted engagement from diverse groups than traditional passive recruitment methods.^9^ However, technology adoption within the same country, for instance in the UK, as well as across other countries, remains variable. For example, in Colombia, people in rural areas may be less motivated to use health‐care technology due to both a lack of technological understanding and poor connectivity, while in Brazil underserved populations tend to be more open to receiving care locally through public services and civil society organizations than remotely, through telehealth systems, for instance.

What is more, the appropriateness of cognitive or reading tasks can be heavily influenced by the literacy levels of participants. This variation in literacy can skew results, as individuals with higher literacy may perform differently on certain tasks compared to those with lower literacy levels, potentially leading to biased outcomes in the analysis of speech biomarkers, which is often the case in analyses conducted in the Colombian population.^45^ A sustainable system of care for brain health that includes diverse populations means not only equipping local health‐care workers with remote screening tools but also raising awareness around healthy aging in local communities and the benefits of early engagement with screening or monitoring programs for neurodegenerative disease.

## 6 TRANSLATING RESEARCH ON SPEECH BIOMARKERS TO CLINICAL SETTINGS

Much of the current debate around speech‐based biomarkers for use in clinical settings focuses on the lack of integration between research findings and clinical care, insufficient validation protocols for speech biomarkers, and the lack of normative data to inform clinical decisions. As a result, voice and speech assessments risk being perceived as experimental tools rather than reliable diagnostic aids in clinical settings. Arguably, a key barrier to clinical utility is limited understanding around the *output* of what speech‐based assessments mean, especially compared to commonly used tools in primary or secondary care settings. A feasible approach to using speech‐based biomarkers in clinical settings is therefore deploying clinician‐friendly digital tools with automatic scoring and presentation of results in relation to normative data that are clear to interpret. Any patient‐facing computer applications would need to be designed with patient and public involvement to aid adoption of this type of health‐care technology among the target disease population.

Experience in Colombia has shown that patients value easy‐to‐understand immediate feedback;^45^ however, varying and unknown acoustic conditions where recordings are made may generate a technical barrier for consistent output and interpretation of the results. We present some of the practical learnings in remote speech data collection and possible solutions in Table 1. Our experience in the UK suggests that clinician training in interpreting output from health‐care technology, such as remote speech collections tools, should be as quick and easy as possible to fit into clinical workflows. Importantly, any output from such digital tools would need to offer meaningful information to the clinician regarding risk of disease or progression trajectory on an individual level. We also see this in the United States, where health‐care professionals’ unfamiliarity with speech biomarker technology adds complexity, particularly where its benefits are unclear in the absence of or limited access to disease‐modifying therapies.

**TABLE 1:** Using speech assessments in remote monitoring of cognition.

Practical issues encountered	Mitigation strategy
Researchers do not commit to data sharing from the outset; that is, no data sharing statement in the participant informed consent form	Include an explanation about data sharing in participant information sheets and ask consent for data sharing (even if consenting to this point is optional).
Opt‐in consent results in low recruitment numbers	Design study protocols with opt‐out consent where appropriate.
Participants complete the remote study assessment multiple times, creating multiple entries for one data point	Consider disabling the data collection for each user ID after the assessment has been completed for a limited period, for example, 1 month (but have the platform available again for future follow‐up visits). Send an e‐mail confirmation after a remote visit has been completed to avoid the participant getting confused if they have completed the assessment already.
Participants complete the remote study assessment outside the visit window	Define data capture timeframe (e.g., 1 month after issuing invitation) or account for varying visit windows in longitudinal analysis (e.g., dynamic structural equation models).
Multiple participants may use the same e‐mail address (e.g., a couple sharing an account), creating two entries from different individuals with the same associated e‐mail address	Verify all entries with e‐mail address and additional identifiers (e.g., initials, date of birth, phone number).
Participants struggle with basic software navigation	Co‐develop simple set‐up guides with patient or public involvement; provide technical support; identify digital champions within remote and rural communities.
Someone other than the participant performs the remote tests (e.g., grandchildren)	Implement technological safeguards, such as voice‐identification algorithms. Co‐design the data collection tools and instructions with input from the target population and culture. Educate health‐care professionals about remote monitoring and how they can explain the importance of data collection to patients under their care.
Participants find instructions hard to follow	Adapt instructions to patients’ backgrounds to ensure accessibility and comprehension. Be flexible with the battery you share with each patient depending on their ability/background.
Lack of acoustic consistency in the collected data	Ask participants to read a standardized paragraph to establish baseline acoustics and noise profiles. Encourage consistent recording conditions (hand out devices if possible to keep settings the same). Collect information about the device used (e.g., sampling rate).
Acoustic descriptors, particularly those related to spectral information, reflect the recording environment rather than to the patient's health state	Apply additional pre‐processing such as segmentation of the speech data to ensure only context‐independent acoustic descriptors for the patient are included.
Issues with content quality when participants fail to follow specific prompts or instructions	Co‐design task prompts with the target population to ensure the prompt is interpreted in a way that produces the appropriate response; pilot the design prompts.
No rural/remote hospitals	Partner urban hospitals with regional clinics, using speech biomarkers as an early screening tool where other options are limited.
People prefer in‐person health care	Integrate speech capture into routine doctor visits instead of relying solely on remote methods; combine digital delivery with in‐person delivery of remote data collection and care.

Colombia's health‐care system is confronting similar challenges, in which professionals in rural and remote regions, where many of the individuals who carry genetic mutations reside, are not familiar with the use of speech‐based tools, which could greatly aid in early detection and ongoing management of dementia. Similarly in Greece, where specialized services are located mainly in the largest cities (Athens and Thessaloniki), a major barrier is the lack of awareness and training of general practitioners. Simple training programs could help integrate this technology into routine care.

## 7 INCORPORATING SPEECH TECHNOLOGY INTO CLINICAL PRACTICE

In clinical practice, remote speech assessments can support traditional care, flag individuals who need further testing based on their ongoing monitoring, triage, and integration into workflows without requiring additional appointments at the clinic. Clinician training on interpretation of speech assessments and the integration of these outputs that go beyond raw scores into electronic health records should aid diagnosis and treatment planning. The US DementiaBank team is developing Grand Rounds online resources to provide education to students and clinicians on discourse‐informed assessment and treatment that leverage the free educational resources available through TalkBank. Once clinicians feel comfortable to communicate speech‐based assessment results on an individual basis (rather than just averages), remote tools could offer an additional source of certainty for the clinician regarding accuracy of diagnosis/prognosis and for the person with disease.

It is also critical to involve family members and provide education on speech biomarkers’ importance, so family members can facilitate conversations in familiar interactions that generate spontaneous speech. This is particularly relevant in the UK, where most studies run by research institutions include care partner involvement for monitoring brain health. Evidence from research settings suggest that a dyad approach in which both the person with disease and their partner are involved may be preferable to some individuals when it comes to speech biomarker assessments.^46^ Similarly in Greece, family may play an even greater role in dementia care because many people with AD rely on relatives for support. Therefore, involving caregivers in speech assessments could make the process more natural and improve data quality. Experience in the UK health system also suggests that opt‐out rather than opt‐in consent for speech data collection with transparent communication about interpreting output of speech data may yield better engagement than opt‐in consents.

In countries such as Brazil and Colombia, which cover large geographic areas and are characterized by uneven population distributions and regional diversity, remote assessment is also seen as an opportunity for better understanding of the epidemiological profile of ADRD. However, there may be operational difficulties in individuals completing speech‐based assessments remotely, despite providing both voice and text instructions. Across the settings with which the authors of this paper have experience, we commonly observe that participants struggle with basic software navigation, and after multiple failed attempts, many simply give up in frustration.

On occasions when individuals have been unable to complete the tasks themselves, individuals have asked their grandchildren to perform the remote tests—a well‐intentioned but problematic solution that compromises data integrity. This type of data collection therefore needs careful consideration around privacy and task authenticity in shared living environments. Even when limited to assisting the individual taking the test, dependence on grandchildren setting up the assessment has introduced a bias and can affect the data. In Colombia, when the focus is on early detection and monitoring of patients,^47^ it can be hard for people with an ADRD diagnosis to follow specific instructions to perform appropriate data collection remotely,^45^ compounded by noisy environments, which can degrade the quality of speech recordings. Additionally, in the UK we have observed that many senior couples may share a single e‐mail address between two people, which adds a layer of complication to remote data collection, particularly if platforms are designed to prevent duplicate entries with the same e‐mail address to prevent the same participant accidentally completing the assessment more than once. These practical experiences underline a need for simple, user‐friendly platforms.

## 8 PATHWAY TOWARD VALIDATION OF SPEECH BIOMARKERS

One of the limitations of speech biomarkers in AD and their limited uptake in clinical settings is a lack of clinical validation. Development of clinically meaningful insights from speech biomarkers would require interdisciplinary collaboration between data analysts and clinical experts, robust data collection at a large scale, and alignment with gold‐standard neurodegeneration metrics. Validation of speech biomarkers should involve the use of established biomarkers, such as amyloid positron emission tomography or cerebrospinal fluid analysis, as a reference standard, though there remains uncertainty around the psychometric properties even with gold standard biomarker assessments, primarily regarding specificity of the underlying etiology of disease pathology.^48^

While standardization of how data are collected or harmonized across existing data sets would ensure generalizability across populations,^49^ validation strategies should also be shaped by cultural contexts. In Taiwan, for example, where there is a strong cultural preference to receive health care in person, a window of engagement has been during the extended waiting times for in‐person appointments when speech data could be collected. In the United States, a move toward decentralized trials has resulted in an increase in mobile‐enabled tools to reach diverse populations. In Brazil, the structure of the public health‐care system also favors decentralization. Validating speech biomarkers for AD in Greece and Colombia comes with challenges but also exciting opportunities that complement traditional assessment methods. Given the scarcity of non‐English datasets, collecting language‐specific data is crucial for accurate modelling and future results^50^.

## 9 EMPOWERING INDIVIDUALS TO MAKE DECISIONS ABOUT THEIR BRAIN HEALTH

In a real‐world setting, difficulties in language and communication may be dismissed as part of normal aging, or “stress related” based on our experience, for example, in Taiwan. However, robust speech assessments in AD and comparison to normative data may result in high ecological validity and meaningful insights regarding the likely underlying causes of subtle changes observed in real‐life speech. Additionally, knowledge of underlying causes may lead to greater engagement with clinical services and monitoring of brain health. Despite the barriers outlined above in adopting new health technologies, we speculate that *willingness* to engage with speech‐based assessments might be due to these tools’ alignment with everyday behaviors, and that this should be leveraged for positive engagement.

Evidence around disclosure of early brain disease like MCI suggests clinicians require better tools to identify, disclose, and monitor clinical syndrome and neurodegenerative disease diagnoses.^51^ In this regard, insights and metrics from remotely conducted speech‐based monitoring could be used to support meaningful conversations about disease status and follow‐up care between the clinician and the person engaging with services. We note that output from cognitive assessments using speech‐based assessments should be explained to the person in the same manner as physical measurements (blood pressure, cholesterol levels, etc.), with interpretation of trends over time and of what these indicate, predict, and correlate with. It is critical that detection and monitoring methods enable a person to make informed decisions, whether that is the decision to enroll in clinical trials, consider taking newly approved treatments, or modify lifestyle to reduce risk of further decline. In cultural contexts globally, where subtle speech changes are often normalized in older age, speech biomarkers shift perceptions from accepting certain decline to empowering individuals to maintain cognitive health and encourage early intervention.

There is no consensus on whether the person completing speech‐based assessments remotely should have access to their own ongoing monitoring results, or indeed, whether their care partner should have such access. Although there are concerns that cognitive health assessments may highlight declines that could negatively impact patients’ emotional well‐being if appropriate clinical management is not in place, our experience in Colombia and Brazil has shown that older adults *want* to know about their health/brain state and are likely to look for technology to provide them with feedback and further recommendations to keep improving their health. Indeed, empowering individuals with knowledge of their health seems most appropriate, especially as poor performance may flag either a separate modifiable condition or allow individuals to take action to slow down progression of the neurodegenerative process. In those cases, health technology could be used to create new speech/language tasks that motivate keeping mentally active. However, for such a technology to be used worldwide, it is necessary to solve the problem of creating robust AI foundation models in different languages, especially adapted to different neurological conditions like ADRD.

## 10 FUTURE DIRECTIONS

The focus for research and development programs advancing speech technologies in neurodegenerative disease should be on collecting data from diverse populations, and ensuring these data are widely accessible to speech biomarker researchers. Data sharing should include not only the development of technical means of privacy protection adequate for protection of speech data, but also ethical and legal frameworks for data collection and sharing. Addressing these issues is crucial to ensure current barriers to biomarker validation are overcome, and subsequent translation to clinical practice in enabled. To date, no large‐scale studies exist on the use of speech as a means of cognitive assessment at a public and global health level. There is a need for larger, global health research programs to be implemented to investigate the role of speech in promoting effective public health approaches to meet the WHO ADRD diagnosis targets and ultimately empowering individuals to make decisions about their brain health.

## 11 CONCLUSION

Speech biomarkers have great potential as a cost‐effective and non‐invasive method for engaging individuals remotely for the detection and monitoring of neurodegenerative disease behavioral markers. In this paper, we draw on our experience from a range of locations across the world to highlight the diagnostic potential, limitations, and future directions for the speech biomarker field in neurodegenerative disease. While our paper does not intend to be exhaustive of these areas, we illustrate that there are shared challenges in the adoption of speech biomarkers in monitoring cognition and brain health. The main areas that need solutions include acquiring multilingual data sets, standardization in data collection and analysis, privacy (accounting for varying regional regulatory frameworks), and interdisciplinary collaboration. In particular, interdisciplinary collaboration should include not only disciplines such as machine learning, signal processing, neurology, and speech and language therapy, but also linguistics and cultural anthropology to ensure that local languages and cultures are appropriately addressed. Finally, a consensus on the clinical interpretation of speech biomarker data is also needed.

## CONFLICT OF INTEREST STATEMENT

The authors declare no conflicts of interest. Author disclosures are available in the Supporting Information.

## Supporting information



Supporting Information
